# Chronic Morbidity in Patients with Endometrial Cancer who Received Adjuvant Radiotherapy

**DOI:** 10.7759/cureus.6325

**Published:** 2019-12-08

**Authors:** Dharely Raquel Cid Sánchez, Rodolfo Rivas Ruiz, Odilon Félix Quijano Castro, Onix Garay Villar, Alejandro R Camacho

**Affiliations:** 1 Radiation Oncology, Oncology Hospital of National Medical Center, Mexico City, MEX; 2 Pediatrics, XXI Century National Medical Center, Mexico City, MEX; 3 Oncology, XXI Century National Medical Center, Mexico City, MEX; 4 Radiation Oncology, Mexican Institute of Social Security, Mexico City, MEX; 5 Radiation Oncology, National Institute of Neurology and Neurosurgery, Mexico City, MEX

**Keywords:** endometrial cancer, high intermediate risk, external beam radiation therapy, brachytherapy, chronic toxicity

## Abstract

Background

Endometrial cancer is the second gynecological neoplasm in our country. The standard treatment is surgery, followed by radiation therapy or chemotherapy according to the stage.

Aim

The purpose of this study was to determine the frequency and type of chronic morbidity in patients with high to intermediate risk of endometrial cancer according to European Society for Medical Oncology (ESMO) 2016, treated with radiotherapy in its modality of external beam radiation therapy and/or brachytherapy in the Oncology Hospital of National Medical Center XXI Century from 2012 to 2016.

Methods

This is a longitudinal, observational, retrospective study of 37 patients diagnosed with high to intermediate risk of endometrial cancer, who received external beam radiation therapy and/or high-rate brachytherapy and follow-up in the unit.

Results

Up to 87% of the patients, who met the criteria of high to intermediate risk, received adjuvant treatment with radiotherapy; 44% brachytherapy, 43% teletherapy, and 13% of patients did not receive adjuvant treatment. Seventy percent presented toxicity associated with radiotherapy, with 65% of the cases being grade 1 and 2 and 5% of cases grade 3; there was no grade 4 toxicity. Regarding the site, the digestive tube occupied the first place with 38% of the cases. The univariate and multivariate analyses showed that age over 65 years is the only factor with statistical significance to develop chronic morbidity.

Conclusion

Age >65 years is the independent risk factor associated with the development of chronic toxicity.

## Introduction

Endometrial cancer is the first gynecological cancer in frequency and the fourth most common cancer in women in developed countries, with an incidence of 24.3 cases in 100,000 women and a mortality rate of 4.3 per 100,000 women per year in the United States [1-3]. 

In developing countries, such as Mexico, it is the second gynecological neoplasm, only behind cervical-uterine cancer, in women older than 20 years [3].

Regarding the increase in life expectancy, the use of tamoxifen and obesity has significantly increased its incidence. Typically, it occurs in postmenopausal women aged 55 to 85 years, with an incidence of 95 in 100,000 in those aged 65 [4]. 

As for the histological type, endometrioid adenocarcinoma is the most common, being present in more than 75% of cases, followed by serous adenocarcinoma (5% to 10%) and mucinous (1% to 5%). Histologic variants other than adenocarcinoma (squamous, transitional cells, small cells, and undifferentiated) represent 1% [5]. 

Based on their biological, clinical and epidemiological characteristics, two types have historically been distinguished:

Type I: It is the most common, derived from chronic exposure to estrogens, predominantly in young, obese and perimenopausal women, associated with endometrial hyperplasia with atypia as a premalignant lesion. It is characterized by slow growth, endometrioid histological type, low grade, superficial invasion depth, and alterations in PTEN or K-RAS genes.

Type II: It is associated with aggressive disease, not related to estrogen stimulation, rapid growth, high degree, deep invasion, p53 mutation in 90% of the cases, and aggressive histological variants (papillary serous, clear cells, and carcinosarcomas) [6]. 

The clinical presentation involves transvaginal bleeding, which is present in up to 90% of postmenopausal patients at diagnosis, although the incidence of endometrial cancer in this group of women is only 10% to 15% [7]. 

Currently, the treatment of choice is surgery consisting in total abdominal hysterectomy, bilateral salpingo-oophorectomy with or without lymphadenectomy in early stage, and radical hysterectomy for an advanced disease can be performed by conventional laparotomy, laparoscopic route, and in specialized centers, robotic laparoscopic. Once the surgery has been performed, the need for adjuvants will be based on the final histopathological report. The surgical specimen will provide data such as depth of invasion, lymphovascular invasion, tumor size, and extrauterine disease (lymphadenectomy and peritoneum biopsy). Therefore, the risk of recurrence of each patient can be estimated, and then a risk-benefit analysis of adjuvant treatment can be performed [8-9]. 

Regarding the adjuvant treatment, different prognostic factors have been established throughout history, which may vary somewhat depending on the school (depth of invasion to the myometrium, age of the patient, lymphovascular invasion, histological grade). The most accepted risk factors are derived from the Post-operative Radiation Therapy in Endometrial Carcinoma (PORTEC) and the Gynecologic Oncology Group (GOG) studies, which are those published by the European Society for Medical Oncology (ESMO) in its 2016 update.

The PORTEC 1 study demonstrated the benefit of radiotherapy after surgery, in terms of local control for the high-to-intermediate risk group, at the cost of moderate toxicity, such that in 2010, the PORTEC 2 study was published and demonstrated the non-inferiority, in local control, of the treatment with brachytherapy with respect to teletherapy as adjuvant management, but a significant decrease in gastrointestinal toxicity of 12.6% vs. 53.3% [10-11].

## Materials and methods

This study received an endorsement from the Research and Bioethics Committee of the unit; a longitudinal, observational, and retrospective study was conducted. In all, 300 patients diagnosed with endometrial cancer were treated from January 2012 to January 2016, of which only 37 met intermediate-high risk criteria according to ESMO 2016. It was essential that patients were followed up in the hospital; patients who received surgical and adjuvant management in the unit but were monitored in another center were excluded. Demographic characteristics of patients with high-intermediate risk endometrial cancer are shown in Table 1.

**Table 1:** Demographic characteristics of patients with high-intermediate risk endometrial cancer ECOG: Eastern Cooperative Oncology Group, functional state

Variable	Value	N (%)
Age	>60 years	13 (35)
	<60 years	24 (65)
ECOG	0	18 (48)
	1	17 (46)
	2	2 (6)
	3	0 (0)
	4	0 (0)
Comorbidity	Irritable Bowel	17 (46)
	Diabetes Mellitus 2	8 (22)
	Systemic Arterial Hypertension	10 (27)
	Other	2 (5)
Clinical stage	IA	10 (27)
	IB	27 (73)
Histological grade	1	4 (11)
	2	28 (76)
	3	5 (13)
Lymphovascular invasion	Yes	21 (57)
	No	16 (43)
Body mass index	Normal	9 (24)
	Obesity	18 (49)
	Overweight	10 (27)

To measure the degree of toxicity, the Common Terminology Criteria for Adverse Events version 4 (CTCAE v.4) scale was used, where, in general, grade 1 is a mild adverse effect, grade 2 moderate adverse effect, grade 3 severe adverse effect, grade 4 life-threatening adverse effect, and grade 5 death [12].

Statistical analysis was performed with measures of central tendency and dispersion, including mean, median, standard deviation, and interquartile ranges, according to the type of distribution. Frequencies and percentages were also calculated.

## Results

Of the 300 medical records analyzed, according to the ESMO 2016 risk groups, the distribution was as follows: 96 patients were at low risk, 44 patients intermediate risk, 37 patients intermediate high risk, 82 patients high risk, and 22 patients advanced disease. It is important to mention that there were 17 medical records opened for diagnosis of endometrial cancer whose diagnosis was different. Therefore, the population analyzed in this research study consisted of 37 patients who met the high-to-intermediate risk criteria according to the ESMO 2016 and who were followed up at the National Medical Center XXI Century.

The average age was 63 years, with a minimum age of 38 and a maximum age of 82. Regarding the functional status, 48% of the population had ECOG 0, 46% ECOG 1, 6% ECOG 2; all patients had an adequate functional status.

Up to 35% of patients had some comorbidity, the most common ones being irritable bowel syndrome in six patients (46%), diabetes mellitus type 2 in three patients (22%), systemic arterial hypertension in three patients (22%), and Lynch syndrome in one patient (10%).

Regarding the clinical stage, up to 73% of the patients presented clinical stage IB and 27% IA, with respect to the histological grade, grade 1-2 was found in 73% of the samples, and grade 3 in 27%. Likewise, the percentage of Lymphovascular invasion was 57%.

To conclude with the characteristics of the population, we mention that the majority of patients were overweight or obese, 49% and 27%, respectively.

Up to 87% of patients who met the high-to-intermediate risk criteria according to the ESMO 2016 received adjuvant radiotherapy; 57% received brachytherapy and 43% teletherapy. Regarding the frequency of chronic morbidity, it was present in 70% of the patients, being grade 1-2 in 75% of cases and grade 3 only in 5%; no grade 4 toxicity was present.

Regarding the site of chronic morbidity, the digestive tract ranked first with 38% of cases, followed by vaginal mucosa (16%), skin (11%), and bladder (5%).

The univariate analysis showed that from all the variables analyzed, age was the only one that presented statistical significance for the development of chronic morbidity, which continued to be present in the multivariate model. Results of univariate and multivariate analyses are shown in Tables 2-3.

**Table 2:** Univariate analysis LVI, lymphovascular invasion; OR, odds ratio

	Morbidity			CI 95%	
Variable	Yes n=24 (%)	No n=13 (%)	OR	Min	Max
Obesity	18	10	0.9	0.184	4.4
Grade 1-2	12	20	Reference		
Grade 3-4	1	4	2.4	0.239	24.003
Stage IA	3	7	Reference		
Stage IB	10	17	0.729	0.153	3.474
LVI	5	16	3.2	0.787	13.017
Age >65 years	16	8	6.02	1.37	26.9

**Table 3:** Multivariate analysis LVI, lymphovascular invasion. OR, odds ratio

	Morbidity			CI 95%	
Variable	Yes n = 24 (%)	No n=13 (%)	OR	Min	Max
Obesity	18	10	0.928	0.164	5.25
Age	16	8	6.074	1.36	26.9
Grade 3-4	1	4	5.66	0.42	75.5
Stage IB	10	17	0.263	0.035	1.97
LVI	5	16	3.76	0.77	18.3

**Figure 1:**
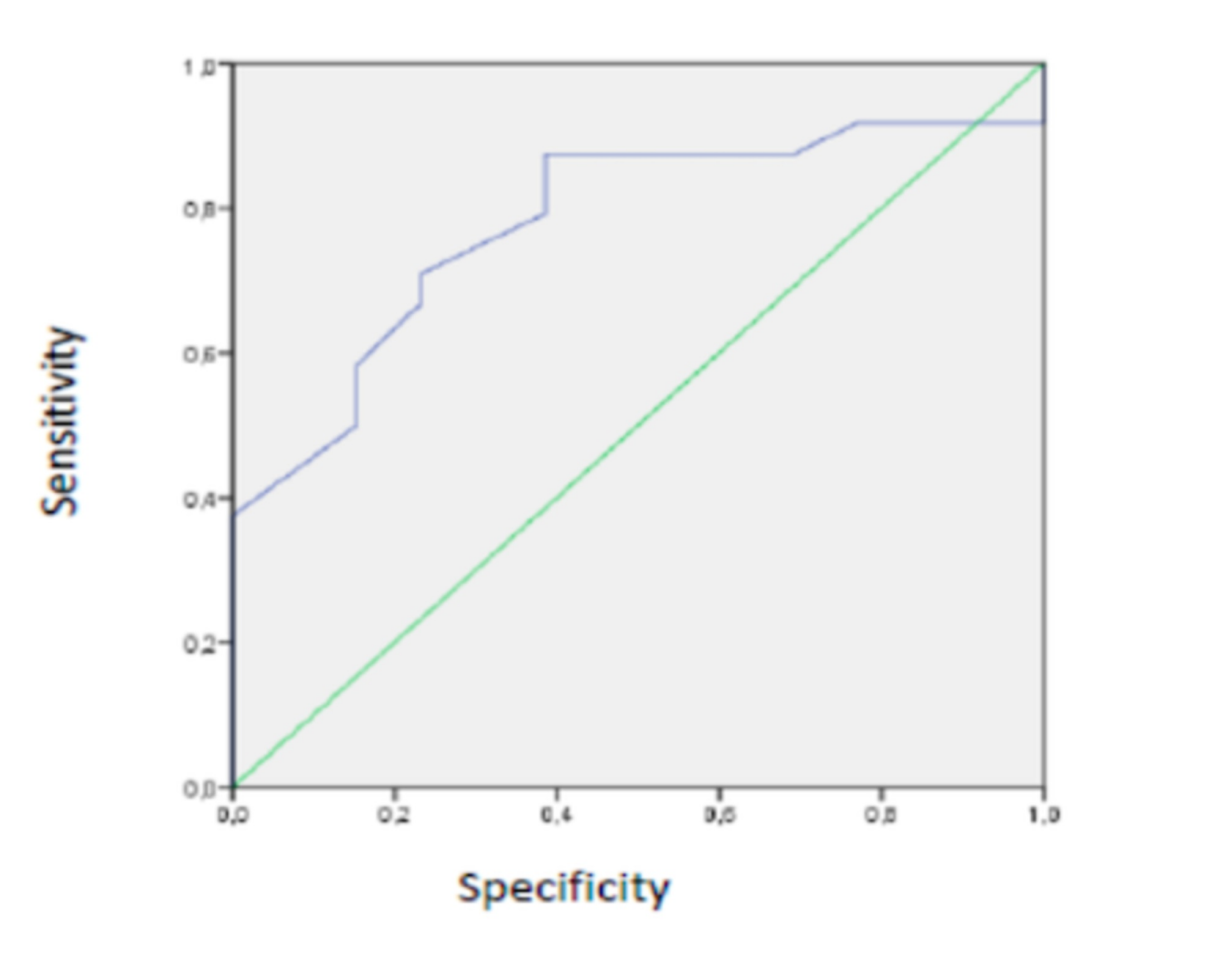
ROC curve ROC, receiver operating characteristic

The receiver operating characteristic (ROC) curve showed that age over 65 years had a sensitivity of 67% with a specificity of 77%, showing the best likelihood ratio of 2.91 in association with chronic comorbidity (Figure 1).

## Discussion

In the current management of patients diagnosed with endometrial cancer, standard treatment continues to be surgical followed by observation, with high chances of cure and recurrence acceptable for low-risk and intermediate-risk patients. However, for patients in the high-intermediate risk, the PORTEC 1 study showed that adjuvant therapy with teletherapy reduced local recurrence at five years from 13.7% to 4.2%, with a benefit still present at 15 years of follow-up. 15.5% vs. 5.8% [10]. 

However, grade 1-2 chronic morbidity was present in a large number of patients significantly decreasing their quality of life, and thus, in 2010, the PORTEC 2 study was published comparing teletherapy vs brachytherapy for adjuvant treatment of the patients with high-intermediate risk of endometrial cancer demonstrating that brachytherapy provided the same local control, with a significant decrease in morbidity 53.8% vs. 12.6%: therefore, it was concluded that the adjuvant treatment of choice for this type of patients was brachytherapy [11]. 

At the National Medical Center XXI Century, the frequency of chronic morbidity secondary to adjuvant radiotherapy in this group of patients was unknown, and this study showed that grade 1-2 chronic morbidity was present in up to 65%, a very high rate for the current standard management. It should not be forgotten that in the present series, up to 43% of the patients received treatment with teletherapy; however, chronic grade 3 toxicity was 5% which is comparable to that referred in the literature [13-15]. 

Regarding the morbidity site, intestinal toxicity was present in 38% of the patients, while 46% of the 37 patients presented irritable bowel as comorbidity; although in the multivariate analysis, no statistical significance was observed between the association of the irritable bowel and chronic toxicity, it showed a trend.

Most importantly, this study found that age over 65 years is the most important prognostic factor for the development of chronic morbidity.

This study was limited in the first instance due to its retrospective nature, the low number of patients who met the high-to-intermediate risk criteria, as well as having different classifications for risk groups in the hospital that come under the high-to-intermediate risk criteria published by ESMO 2016; half of the patients were treated with teletherapy; although it is known that it offers the same local control as brachytherapy, it is not the treatment of choice due to its higher associated toxicity index.

## Conclusions

Considering the limitations of the retrospective design, this study suggests that age over 65 years is the independent risk factor associated with the development of chronic toxicity in patients diagnosed with high-to-intermediate-risk endometrial cancer, who received adjuvant radiotherapy. This fact should be considered as the highest incidence of this pathology is after 65 years of age; hence, we must create strategies that allow us reduce chronic toxicity in our patients.
